# Regeneration of the flatworm *Prosthiostomum siphunculus* (Polycladida, Platyhelminthes)

**DOI:** 10.1007/s00441-020-03302-w

**Published:** 2020-11-07

**Authors:** Tamara Schadt, Veronika Prantl, Alexandra L Grosbusch, Philip Bertemes, Bernhard Egger

**Affiliations:** Research Unit Evolutionary and Developmental Biology, Institute of Zoology, University of Innsbruck, Technikerstr. 25, 6020 Innsbruck, Austria

**Keywords:** Blastema, Flatworm, Polyclad, Proliferation, Regeneration

## Abstract

Fueled by the discovery of head regeneration in triclads (planarians) two and a half centuries ago, flatworms have been the focus of regeneration research. But not all flatworms can regenerate equally well and to obtain a better picture of the characteristics and evolution of regeneration in flatworms other than planarians, the regeneration capacity and stem cell dynamics during regeneration in the flatworm order Polycladida are studied. Here, we show that as long as the brain remained at least partially intact, the polyclad *Prosthiostomum siphunculus* was able to regenerate submarginal eyes, cerebral eyes, pharynx, intestine and sucker. In the complete absence of the brain only wound closure was observed but no regeneration of missing organs. Amputated parts of the brain could not be regenerated. The overall regeneration capacity of *P. siphunculus* is a good fit for category III after a recently established system, in which most polyclads are currently classified. Intact animals showed proliferating cells in front of the brain which is an exception compared with most of the other free-living flatworms that have been observed so far. Proliferating cells could be found within the regeneration blastema, similar to all other flatworm taxa except triclads. No proliferation was observed in epidermis and pharynx. In pulse-chase experiments, the chased cells were found in all regenerated tissues and thereby shown to differentiate and migrate to replace the structures lost upon amputation.

**Electronic supplementary material:**

The online version of this article (10.1007/s00441-020-03302-w) contains supplementary material, which is available to authorized users.

## Background

The discovery of regenerative powers in free-living flatworms (‘Turbellaria’) dates back over 200 years, when only a small piece of a triclad’s body was found to regenerate a complete organism (Pallas 1767-1780). Members of the taxon Tricladida are also referred to as planarians. Their regenerative capabilities are so remarkable that in an early study, the triclad *Polycelis nigra* was referred to as being ‘almost immortal under the edge of the knife’ (Dalyell 1814). The regeneration process in flatworms starts with contraction of ring muscles in the region of the injured tissue (Cebrià et al. 1997; Egger et al. 2009a; Girstmair et al. 2014). This contraction is followed by morphallactic remodeling of old tissues to close the wound site. Wound closure is succeeded by epimorphosis, an extensive cell proliferation event at the wound site and the resulting blastema formation (Reddien and Alvarado 2004). Tricladida show little to no cell proliferation inside this regeneration blastema but rather outside at the boundary of the wound site (Saló and Baguñà 1984). In this regard, Tricladida differ from any other studied free-living flatworm taxa, which all show a high abundance of proliferating cells within the regeneration blastema, which suggests that this proliferation-free blastema is an apomorphy of the Tricladida (Egger et al. 2009a; Dirks et al. 2012; Girstmair et al. 2014; Bertemes et al. 2020). The only proliferating cells in flatworms are undifferentiated mesenchymal cells called neoblasts. These pluripotent stem cells are capable of differentiating into all other cell types from any germ layer (Wagner et al. 2011). All rhabditophoran flatworms (Rhabditophora, by far the largest group of flatworms) show a specific stem cell system, with ectodermally derived tissues being devoid of proliferating cells (Egger et al. 2009b; Lapraz et al. 2013; Okano et al. 2015).

Like triclads, polyclads belong to the free-living flatworms. Polyclads are macroscopic, almost exclusively marine rhabditophoran flatworms. They are divided into Cotylea (350 described species), predominantly presenting a ventral sucker posterior to the female copulatory opening and Acotylea (450 described species) without such an organ (Lang 1884; Martín-Durán and Egger 2012; Dittmann et al. 2019). Name giving for the taxon Polycladida is the gut with its many ramifications (Lang 1884). While planarians have been the focus of many detailed regeneration studies, polyclads have not been studied to this extent. To this date, only few studies focus on polyclad regeneration in high detail, with acotylean species being much more often the focus of such studies than cotylean species (Bertemes et al. 2020). Hence, not much is known about the regeneration capacity of cotylean species. Until now, the regeneration capacity of only nine cotylean species has been mentioned in previous studies. Out of these, only two species were studied in high detail regarding their regeneration capacities: *Thysanozoon brocchi* (Monti 1900; Levetzow 1939) and *Theama mediterranea* (Child 1905b; 1905c; 1905d; Bertemes et al. 2020).

In this study, we look at the regeneration capacity of the cotylean polyclad *Prosthiostomum siphunculus* in detail and answer the following questions: What regeneration capacity does *P. siphunculus* possess? Can it regenerate the major organs, including (parts) of the brain? What can be said about the stem cell dynamics in *P. siphunculus*? Does proliferation take place inside the regeneration blastema?

## Methods

### Specimen collection and animal culture

Animals were sampled in coastal areas of the Mediterranean Sea in Andalusia, Murcia, Granada (Spain), Isola Palmaria (Italy), Calvi (Corsica), Malta, Rovinj and Punat (Croatia) (see Table 1). *Prosthiostomum siphunculus* was collected from the backside of submerged rocks in the intertidal and littoral zone at a sampling depth of not more than 1 m below the water level. In the laboratory, adult specimens of *P. siphunculus* were kept in 3.6% artificial sea water (ASW) at 15 °C or 20 °C in the dark and fed once a week with commercially available live *Tubifex* sp. (cut into small pieces) or thawed adult *Artemia salina.* Animals used for regeneration experiments were not fed starting at least 3 days before amputation (or four to 28 days before fixation) to avoid contamination of the wound site with gut content, to reduce nonspecific background during fluorescent stainings and to avoid contamination of the wound site with gut content.

**Table 1:** Sampling sites of *P. siphunculus* with coordinates and number of collected specimens

Sampling site	Coordinates	Count
Andalusia	36° 04′ 36.5″ N, 5° 25′ 31.6″ W	56
Murcia, Granada	37° 39′ 23.8″ N, 0° 43′ 29.7″ W	99
Isola Palmaria	44° 02′ 52.87″ N, 9° 50′ 27.32″ E	67
Calvi	42° 34′ 00.42″ N, 8° 45′ 25.59″ E	18
Malta	35° 56′ 41.91″ N, 14° 24′ 7.5636″ E	9
Rovinj	45° 4.202″ N, 13° 38.207″ E	94
Punat	45° 00′ 33.2″ N, 14° 37′ 20.6″ E	45

### Amputation

**Fig. 1:**
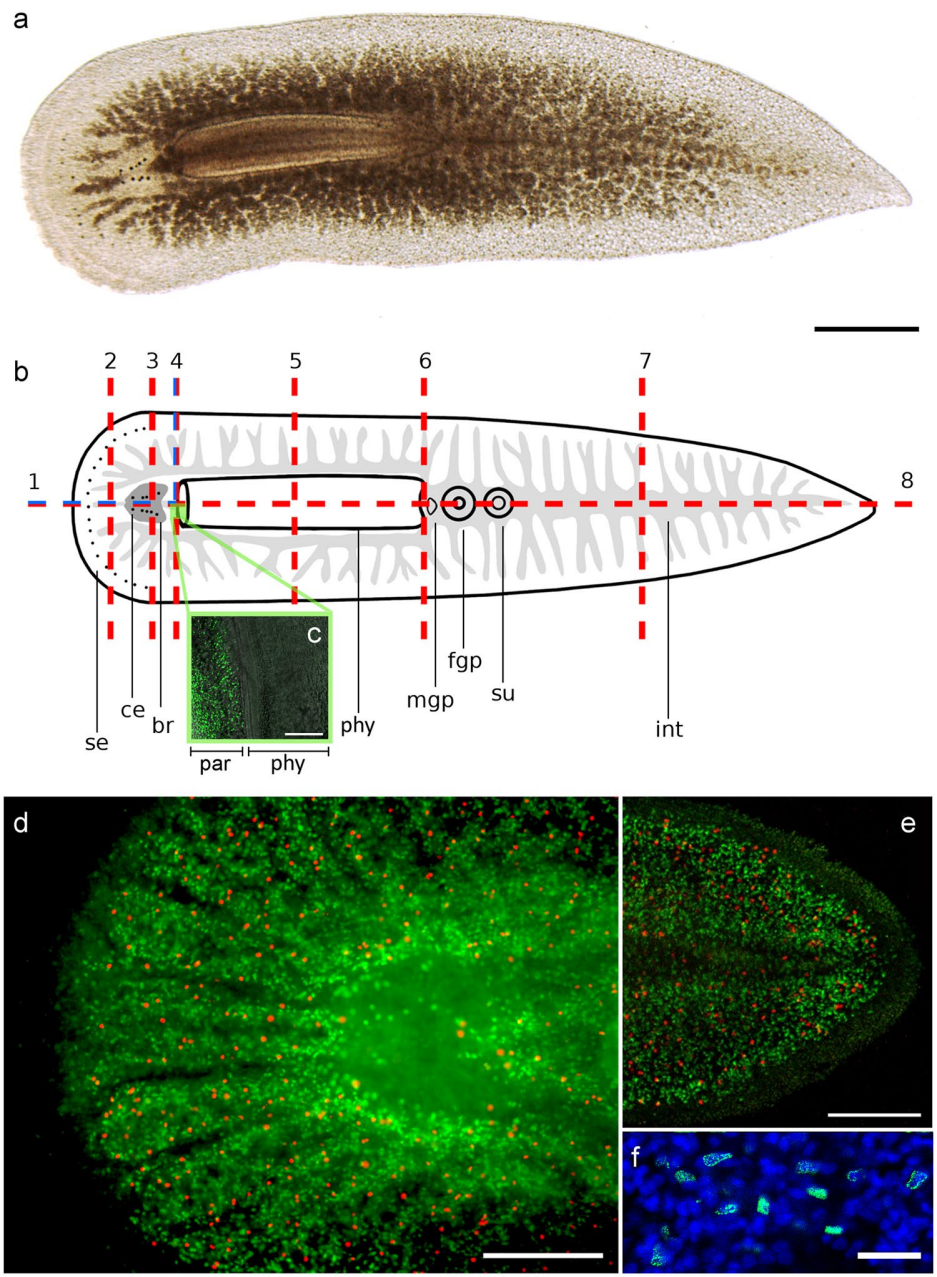
Overview of intact *P. siphunculus* and scheme with amputation levels. **a** Live image of a subadult. **b** Scheme with amputation levels used in the experiments and indication of the main organs. **c–e** Mitotic and S-phase cells visualised by pH3 immunostaining (red) and EdU labelling (green), respectively. **c** EdU-labelled cells in the parenchyme (left), no proliferating cells in the pharynx (right). **d** Head. **e** Tail. **f** Magnification of DAPI- and EdU-labelled cells in blue and green, respectively. Scale bars: **a** 700 µm, **c** 200 µm, **d** 250 µm, **e** 200 µm, **f** 25 µm. Abbreviations: brain (br), cerebral eyes (ce), female genital pore (fgp), intestine (int), male genital pore (mgp), parenchyme (par), pharynx (phy), submarginal eyes (se), sucker (su)

A total of 121 animals were cut at eight different amputation levels (AL) (Fig. 1b). Two of these amputation levels were chosen to examine regeneration of the brain tissue (AL 1 and 3). Pharynx regeneration in anterior pieces was tested with two different levels (AL 4 and 5), while complete pharynx and brain regeneration capacity in posterior pieces was tested with AL 6 and 7. Peripheral body parts were amputated at two other levels (AL 2 and 7) and the last level was longitudinal through the whole animal (AL 8) to test whether regeneration of a lateral half of the animal is possible.

Amputations were made vertically through the brain (AL 1, blue lines, *n *= 10), in front of the brain (AL 2, *n *= 3), horizontally through the brain (AL 3, *n *= 5), right behind the brain (AL 4, red line, *n *= 15), through the pharynx (AL 5, *n *= 3), right behind the pharynx (AL 6, *n *= 4), of the posterior last 30% of the body (AL 7, *n *= 78) and in a last series, longitudinal amputations were made through the entire animal (AL 8, *n *= 3) (Fig. 1b). Regenerates missing anterior structures were termed as anterior regenerates; those missing posterior structures were termed as posterior regenerates.

For AL 1, incisions were made vertically through the brain. A second, horizontal cut was made right afterwards, which resulted in the removal of half the head including half of the brain. The removed piece was then referred to as the smaller regenerate, while the remaining specimen was referred to as the bigger regenerate. Brain regeneration was assessed by live observations and measuring brain dimensions in pictures of live animals. Regenerates of AL 4 and 7 were used for pulse and pulse-chase experiments and referred to as pharynx and tail regenerates respectively.

### Feeding behaviour

To observe feeding behaviour in animals that were cut laterally through the brain the food was placed either right in front of or somewhere inside a Petri dish farther away from the animals. Observations were made using a stereo microscope. Both intact adult *P. siphunculus* (*n *= 3) and regenerates of AL 1, which were missing half the brain (*n *= 10 for bigger pieces, *n *= 1 for smaller pieces), were observed.

### Regeneration

After amputation, animals were kept individually in separate Petri dishes. During the first 3 days after amputation, ASW was exchanged daily. Regenerates of AL 2, 3, 5 and 8 were kept at 20 °C in the dark. Regenerates of AL 1 and 6 were kept at 15 °C. Two different series were made for both AL 4 and 7, where the first series of each was kept at 20 °C and the second at 15 °C to evaluate how temperature affects the speed of regeneration. Regenerates were not fed during the regeneration process. Specimens of AL 1 were fed after 1 month to observe feeding behaviour.

Due to individual differences in regeneration speed among the pharynx regenerates for the pulse and pulse-chase experiments, these specimens were allowed to reach certain regeneration stages before fixation: (1) a blastema was visible in the wounded region, (2) intestinal tissue was visible in the newly formed tissue and (3) a pharynx was visibly regenerated.

For the tail plate regenerates used for pulse and pulse-chase experiments, specific time points were used for fixation: 4, 7 and 11 dpa. Tail plate regenerates were cut once farther above the newly regenerated tissue and only the posterior pieces were fixed and stained.

### EdU labelling

Proliferating cells were labelled with the S-phase marker 5-ethynyl-2′-deoxyudidine (EdU, Invitrogen, USA) in the pulse and pulse-chase experiments. Specimens were labelled with 0.4 mM EdU in ASW in the dark at room temperature (RT) for 60 min. Pulsed specimens were labelled after reaching one of the aforementioned regeneration stages and then fixed. Pulse experiments were conducted to document the proliferating cells at a certain regeneration stage. Pulse-chased specimens were labelled prior to amputation and fixed after they reached one of these regeneration stages to visualise the fate of proliferating S-phase cells at these stages.

Prior to fixation, the animals were anaesthetised with 7.14% MgCl_2_·6H_2_O until they were fully relaxed. Two methods were applied for anaesthesia. In the first, the animal was put on a glass slide with a few drops of liquid surrounding it. Drops of 7.14% MgCl_2_·6H_2_O were slowly added until the animal was relaxed. This method could take up to 1 h. The second method was much more efficient and took only 5–10 min. In this method, the liquid surrounding the specimen was removed. Next, drops of 7.14% MgCl_2_·6H_2_O were added and immediately removed. Animals were unable to fold themselves into balls due to surface tension of the surrounding medium. After successful anaesthesia, they were fixed with thawed 4% formaldehyde (made from paraformaldehyde; Sigma-Aldrich, USA) in 0.1 M PBS for 60 min and afterwards rinsed several times in PBS-T_x_ for another 60 min.

Next, specimens were incubated for 5 min in gradually ascending methanol concentrations (25%, 50%, 75%, 100%) and bleached under bright cold light with a 6% H_2_O_2_ solution in methanol oN to improve the staining quality. After bleaching, they were incubated in gradually descending methanol concentrations (100%, 75%, 50%, 25%) and then once again rinsed several times in PBS-T_x_ for 60 min.tu

The epidermis was permeabilised with 0.1 mg/ml Protease XIV in PBS-T_x_ (Sigma, USA) at 37 °C (up to 40 min). Once the epidermis was ragged, specimens were rinsed again in PBS-T_x_ several times for 60 min and incubated for 60 min in the blocking solution BSA-T_x_.

The specimens were then incubated for 120 min in the freshly prepared Click-iT reaction mix (Invitrogen, USA). Control animals were treated the same and stained as detailed above, except one reagent (CuSO_4_) of the Click-iT reaction mix was omitted.

In one series of pulse experiments, mitotic cells were labelled as well. After the click-iT reaction, the specimens were rinsed with PBS-T_x_ for 60 min and then incubated in BSA-T_x_ for another 60 min. Then, the primary antibody rabbit-anti-pH3 (1:600 in BSA-T_x_; Millipore, USA) was added and kept at 4 °C oN. The next day, the specimens were again rinsed in PBS-T_x_ and blocked in BSA-T_x_ for 60 min. Then, the secondary antibody goat-anti-rabbit-TRITC (1:200 in BSA-T_x_; Invitrogen, USA) was added and kept at RT for 60 min.

In one case, DAPI (Thermo Fisher Scientific, USA) was added at a dilution of 1:1000 (10 µM/ml) in PBS-T_x_ at RT for 60 min after the Click-iT reaction.

All specimens were rinsed with PBS-T_x_ for 3 days and then mounted with VectaShield (Vector Labs, USA).

### Microscopy and visualisation

Amputations were conducted under a Nikon SMZ645 stereo microscope with a razor blade. Pictures of live regenerates were taken with a Leica MZ16F microscope, equipped with either a Leica DFC 490 or a Leica DFC 495 camera (Leica, Germany). A Leica TCS SP5 II confocal microscope was used to make confocal stacks for the EdU pulse and pulse-chase stainings. Stacks were edited with Fiji up to version 1.52i (Schindelin et al. 2012). Fiji was also used to measure pharynx and submarginal eye size. Figures and schemes were made with GIMP version 2.10 (https://gimp.org/) and Inkscape up to version 0.92 (https://inkscape.org/).

### SEM

Specimens of AL 6 were cut once more farther above the newly regenerated tissue and only the posterior pieces were fixed. Specimens were anaesthetised with 7.14% MgCl_2_·6H_2_O, then fixed with 4% formaldehyde in 0.1 M PBS as detailed above, dehydrated in a graded methanol series and then stored at − 20 °C. For further processing, animals were rehydrated in a graded methanol series, transferred in 0.1 M PBS, postfixed for 20 min in 1% OsO_4_ in 0.1 M cacodylate buffer, washed in PBS for 15 min, transferred to distilled water for 4 min and then dehydrated once more in a graded methanol series. Animals were critical point dried with an EMS 850 Critical Point Dryer (Quorum, UK) and coated with 20 nm gold in a CCU-010 coating unit (safematic, Switzerland) and observed on a DSM 950 scanning electron microscope (Zeiss, Germany).

## Results

### Sampling

### Morphology

The length of the collected animals varied from about 6 mm to 3 cm. For experiments, only animals larger than about 1 cm were used. The colour was most often observed to be beige to brownish but yellow pigmentation was also found in some samples. The highly ramified gut ran through the entire body, including the head. At the anterior border, the animals showed a row of submarginal eyes. The number of submarginal eyes varied greatly from animal to animal but the arrangement of the eyes was consistent. The brain lay behind these submarginal eyes in the head of the animal. On top of the encapsulated brain two rows of cerebral eyes were situated. The number of cerebral eyes was more or less equally distributed in these two rows and ranged in samples from 10 to 20 eyes in total. The three-lobed pharynx was located just posterior to the brain and could be protruded. The length of the pharynx made up about 30 ± 7% (*n *= 4) of the entire body length. Posterior to the pharynx were the male and the female genital pores, behind which the sucker was situated. Posterior to the sucker, the tail followed, in which the main trunk of the gut and its smaller peripheral branches could be seen (Fig. 1a).

### Regeneration

#### Amputation vertically through the brain (AL 1)

**Fig. 2:**
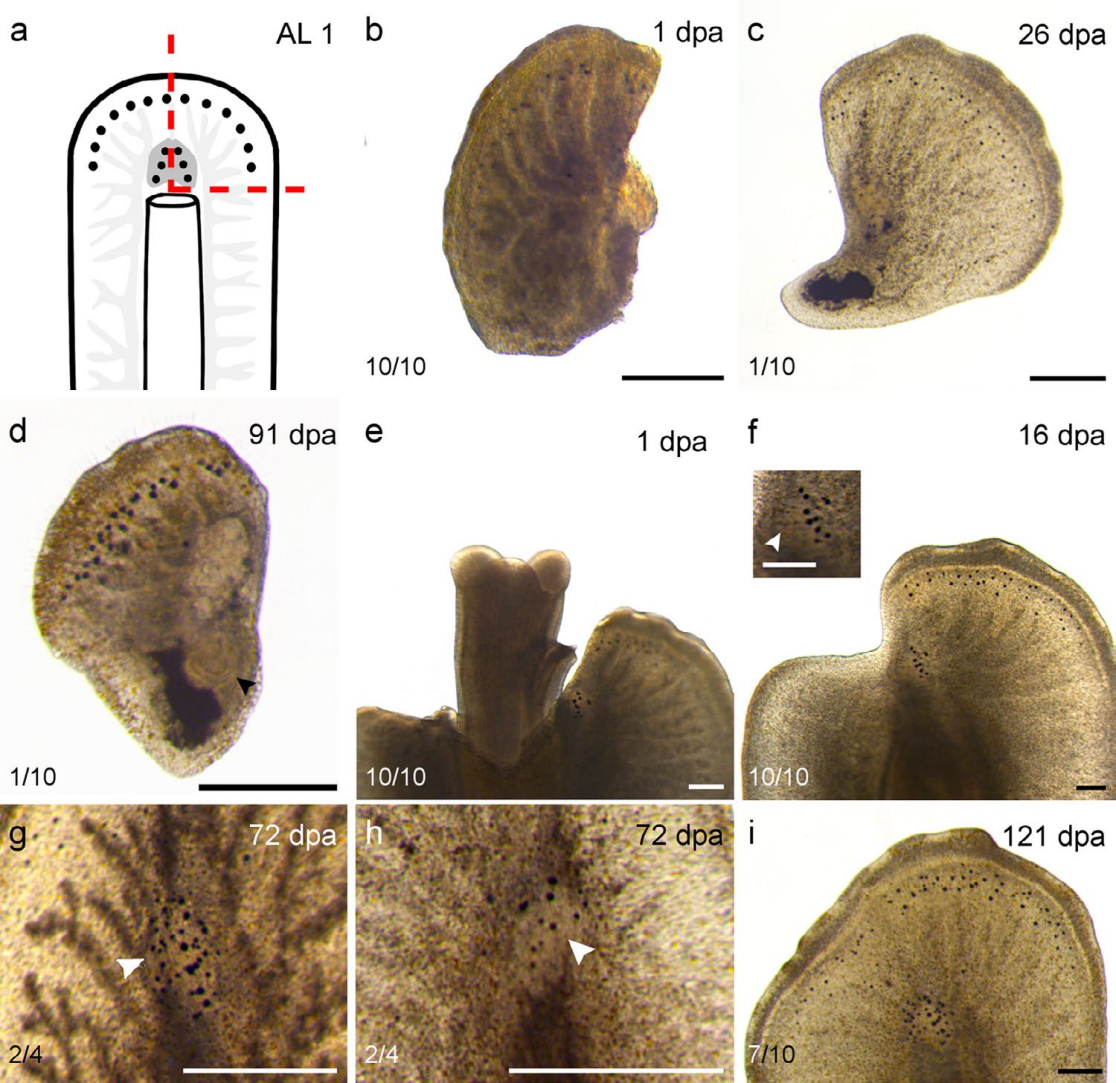
Regenerates of AL 1—vertically through the brain. **a** Scheme with AL 1. **b**–**d** Smaller regenerate (SR). **b** SR with the right half of the brain remaining at 1 dpa. **c** SR closed the wound and reformed its tail tip after 26 days. **d** After 91 days, the SR had regenerated a visible pharynx (arrowhead). **e**–**g** The bigger regenerate (BR) with the right half of the brain remaining. **e** BR with protruded pharynx 1 dpa. **f** Cerebral eyes appeared after 26 days (arrowhead) and visible blastema. **g** BR with more than the right half the brain remaining. New cerebral eyes (arrowhead) regenerated on top of the remaining brain tissue forming two distinct rows with the remaining eyes. **h** BR with only the left half of the brain remaining. New cerebral eyes (arrowhead) regenerated below the remaining eyes on top of the remaining brain tissue. **i** After 121 dpa, the BR had replaced the missing tissue and regenerated cerebral and submarginal eyes. The smaller regenerate (**b**–**d**) is the same individual. The bigger regenerate (**e**–**i**) is the same individual, except the BR with the left brain half remaining shown in **h**. All animals shown from the dorsal side, except **b** and **d**. Scale bars: 200 μm

The smaller regenerates consisted of either the left half of the brain (*n *= 5) or the right half of the brain (*n *= 5) and the surrounding tissues from the outer body wall to the cutting edge (Figs. 1b and 2a). The bigger regenerates made up the rest of the body after half the head was removed (Fig. 2a, e–i).

All smaller regenerates died within the first 2 weeks, except for two specimens. One formed a misshaped blastema and died after 80 dpa (data not shown). The other was found to regenerate a tail tip 26 dpa (Fig. 2c) and a pharynx 91 dpa (Fig. 2d; Table 2) but had shrunk considerably during the regeneration process.

**Table 2:** Summarised lateral regeneration results

**Organs regenerated**	**Within**
Submarginal eyes	22 ± 5 dpa (AL 1, larger piece)
Cerebral eyes	16 ± 2 dpa (AL 1, larger piece)
Pharynx	91 dpa (AL 1, smaller piece)
Tail tip	26 dpa (AL 1, smaller piece)
**Organs not regenerated**	**Within**
Brain	4 months
Whole half of the animal	Died within 2 dpa (AL 8)

All bigger regenerates were able to regenerate cerebral eyes 16 ± 2 dpa (Fig. 2f; Table 2). Four specimens were observed more closely regarding eye regeneration. For two of them, the brain was not cut exactly in half and they had a bit more than half the brain remaining. These two specimens were able to grow back cerebral eyes in two somewhat linear aggregates, with an increase in eye number also above the intact brain half (Fig. 2g). The two specimens that were cleanly cut in half moved their remaining cerebral eyes upward and regenerated new eyes below. Hence, they did not regenerate the cerebral eyes in two distinct rows (Fig. 2h). In all four specimens, it was apparent that cerebral eyes were only regenerated in the presence of underlying brain tissue (Fig. 2g, h). Submarginal eyes regenerated 22 ± 5 dpa with seven submarginal eyes regenerating about every 10 days (data not shown). At the end of the observation period of 4 months, new submarginal eyes had grown back (12.85 ± 2.44 µm) and were significantly (*p* = 1.77E−6, alpha = 0.05, *F*-crit = 4.09) smaller than the remaining submarginal eyes (17.08 ± 2.30 µm) (Fig. 2i; Table 2). For these measurements, 20 submarginal eyes were measured each on the remaining tissue (right side of the animal) and regenerated tissue (left side of the animal).

According to our observations on live animals, none of the specimens were able to regenerate the removed brain tissue during the observation period of 4 months. There was no observed difference between bigger regenerates with the right and those with the left half of the brain remaining (Fig. 2b–i; Table 2).

Compared with animals with an intact brain feeding behaviour was slightly different in the bigger regenerates. Once food (live *Tubifex* sp*.*) was placed near control animals, they immediately moved their head in the direction of the food source, protruded the pharynx and started feeding. The bigger regenerates exhibited problems detecting the direction of the food, if the food was not placed right in front of them. It was observed in three cases that they protruded their pharynx and tried to suck out a piece of their own peripheral body parts instead. They did not resume the regular feeding behaviour observed in intact animals within the observation period of 4 months. No difference in the feeding behaviour of the regenerates was observed between those with the left and those with the right half of the brain remaining. The small regenerate that was able to regenerate a pharynx was not able to resume any kind of visible feeding behaviour.

#### Amputation in front of the brain (AL 2)

**Fig. 3:**
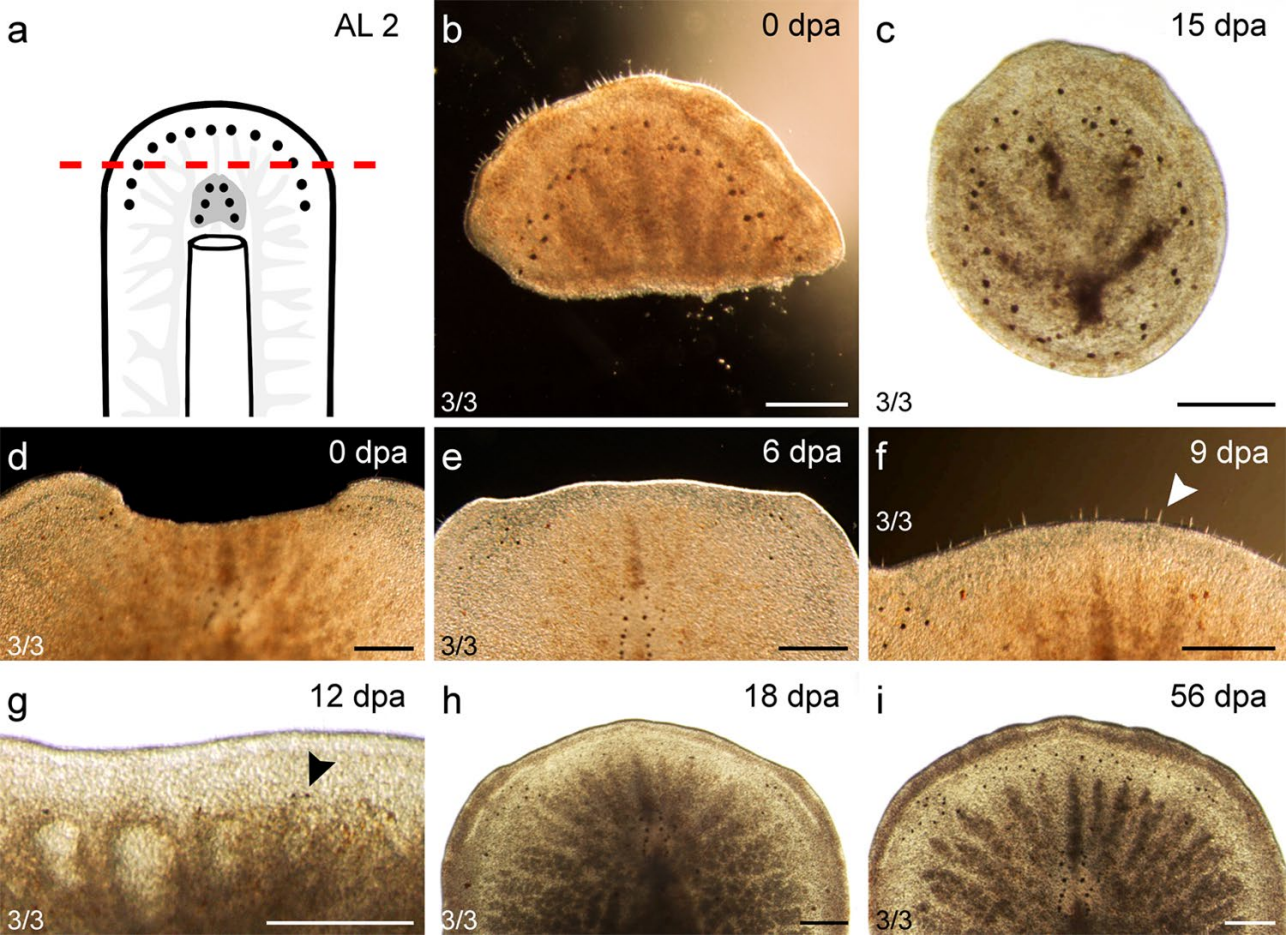
Regenerates of AL 2—in front of the brain. **a** Scheme with AL 2. **b**, **c** The posterior regenerate. **d**, **i** The anterior regenerate. **b** The posterior regenerate right after amputation. **c** After 15 dpa, the posterior regenerate had formed a circular shape and seized to regenerate further. **d** The anterior regenerate right after amputation. **e** The regeneration blastema was well visible 6 dpa. **f** Long sensory cilia had grown back 9 dpa (arrowhead). **g** After 12 dpa, the first regenerated submarginal eyes appeared. **h** After 18 dpa, the regenerated tissue was still well distinguishable from the old tissue. **i** Fifty-six days post amputation, the animal looked similar to before the amputation. Scale bar: 250 µm

The much smaller posterior regenerates formed a circular shape 3 ± 0.6 dpa (*n *= 3) after the cut in front of the brain (Fig. 3a–c). Subsequently, the regeneration process came to a standstill. Dark particles in the intestine accumulated in the posterior end of the animals (Fig. 3c). After 18 ± 5.1 dpa, the posterior regenerates dissolved (Fig. 3b, c; Table 3).

**Table 3:** Summarised posterior regeneration results

**Organs regenerated**	**Within**
Pharynx	12 ± 4.2 dpa (AL 4, 15 °C), 24 ± 4 dpa (AL 4, 20 °C), 12 ± 4.2 dpa (AL 5)
Sucker	14 dpa (AL 6)
Tail plate	10 ± 0.9 dpa (AL 7, 15/20 °C)
**Organs not regenerated**	**Within**
Brain	12 dpa (AL 3)
Genital pores	44 dpa (AL 5), AL 6

In the anterior regenerates (*n *= 3), the area anterior of the brain was missing (Fig. 3d). A blastema became visible 3 ± 0.6 dpa (Fig. 3e) and long sensory cilia grew back 9 ± 1.2 dpa (Fig. 3f; Table 2). The first submarginal eyes appeared 12 ± 2.1 dpa (Fig. 3g; Table 4). From then, about seven submarginal eyes grew back every 10 days until after 5 weeks, no more submarginal eyes were observed to grow back (*n *= 2). At first, the submarginal eyes were very small and hard to distinguish from pigments but grew larger within 3 days and were easy to recognise. After 56 dpa, the difference between intact and amputated anterior regenerates was not visible anymore (Fig. 3i).

**Table 4:** Summarised anterior regeneration results

**Organs regenerated**	**Within**
Submarginal eyes	12 ± 2.1 dpa (AL 2), 9 ± 2.5 dpa (AL 3)
Cerebral eyes	12 ± 1.6 dpa (AL 3)
Sensory cilia	9 ± 1.2 dpa (AL 2)
**Organs not regenerated**	**Within**
Brain	60 dpa (AL 3), 28 dpa (AL 4)
Pharynx	60 dpa (AL 5, AL 6), 35 dpa (AL 7)

#### Horizontal amputation through the brain (AL 3)

**Fig. 4:**
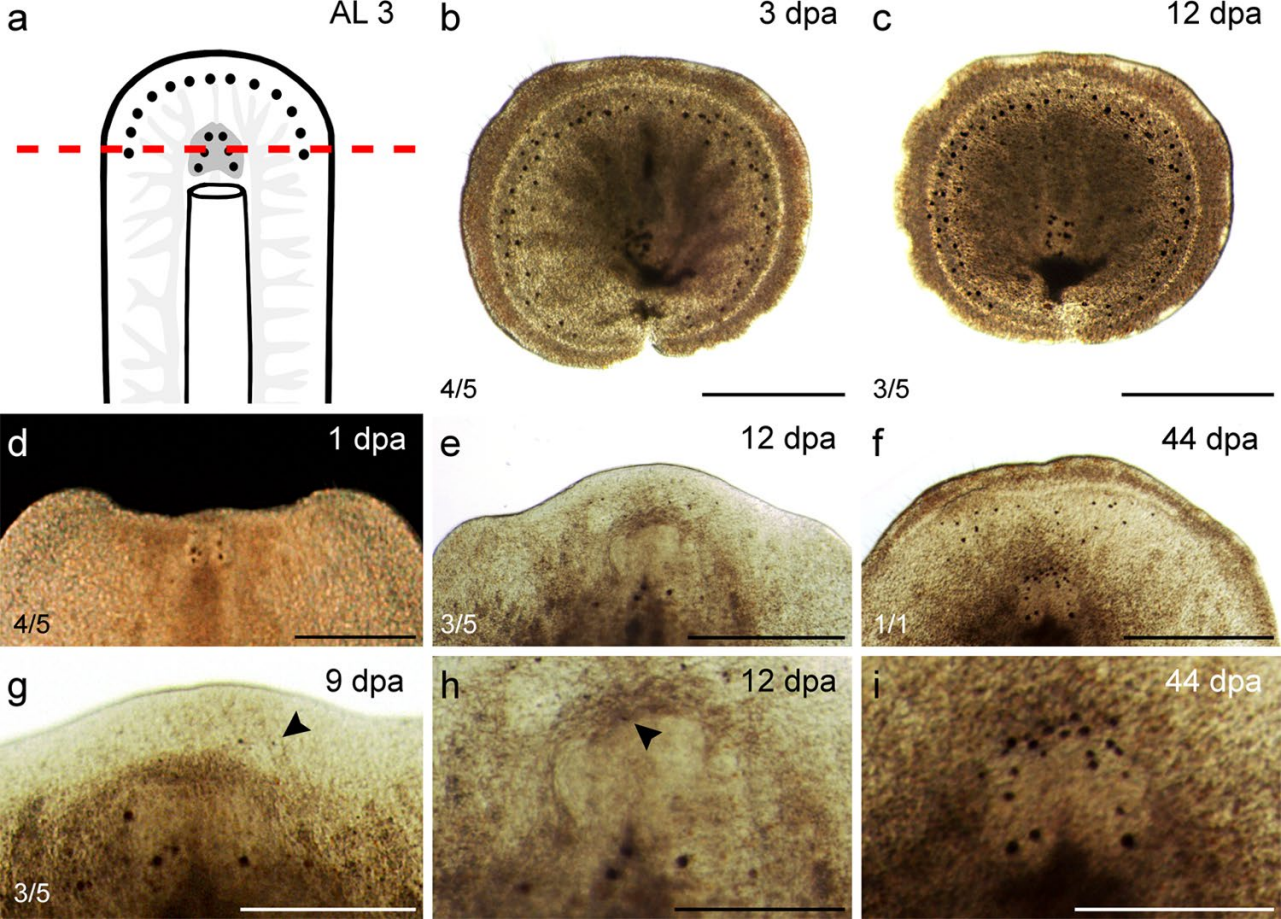
Regenerates of AL 3—horizontally through the brain. **a** Scheme with AL 3. **b**, **c** The posterior regenerate formed a circular shape and dissolved within 24 days without regenerating any missing organs. **d**–**i** The anterior regenerate. **d** The anterior regenerate 1 dpa. **e** The anterior regenerate 12 dpa with some regenerated submarginal eyes and first cerebral eyes (see magnification in **h**). **f** After 44 days, the submarginal eyes had grown in size and cerebral eyes had regenerated. **g** Submarginal eyes (arrowhead) started to regenerate 9 dpa. **h**, **i** Magnifications of **e**, **f** the arrowhead in **h** points at a regenerated cerebral eye. **i** Regenerated cerebral eyes were visible after 44 days. Scale bars: **b**–**f** 500 μm; **g**–**i** 250 μm

The posterior regenerates of AL 3 (*n *= 5) were slightly bigger than those of AL 2, including the upper half of the brain (Fig. 4a). These regenerates contracted the wounded tissue and formed a circular shape within 3 dpa with only a small indentation remaining. After 12 dpa, they formed a circle but did not regenerate any further. Dark particles accumulated in the intestine in the posterior part of the animal. All specimens died within 23 ± 2.3 dpa without regenerating any missing organs (Fig. 4b, c; Table 3).

According to measurements of the brain in images of live regenerates, none of the specimens were able to regenerate the missing brain tissue during the observation period of about 2 months (Fig. 4d–f; Tables 3 and 4). Anterior regenerates of AL 3 were able to regenerate submarginal eyes after 9 ± 2.5 days (Fig. 4g; Table 4). Submarginal eyes grew back at a speed of about seven eyes in 10 days until after 3 weeks, no more submarginal eyes were observed to grow back. Cerebral eyes started growing back 12 ± 1.6 dpa (Fig. 4h; Table 4) and regenerated at roughly the same speed as submarginal eyes (Fig. 4i).

#### Amputation just posterior of the brain (AL 4)

**Fig. 5:**
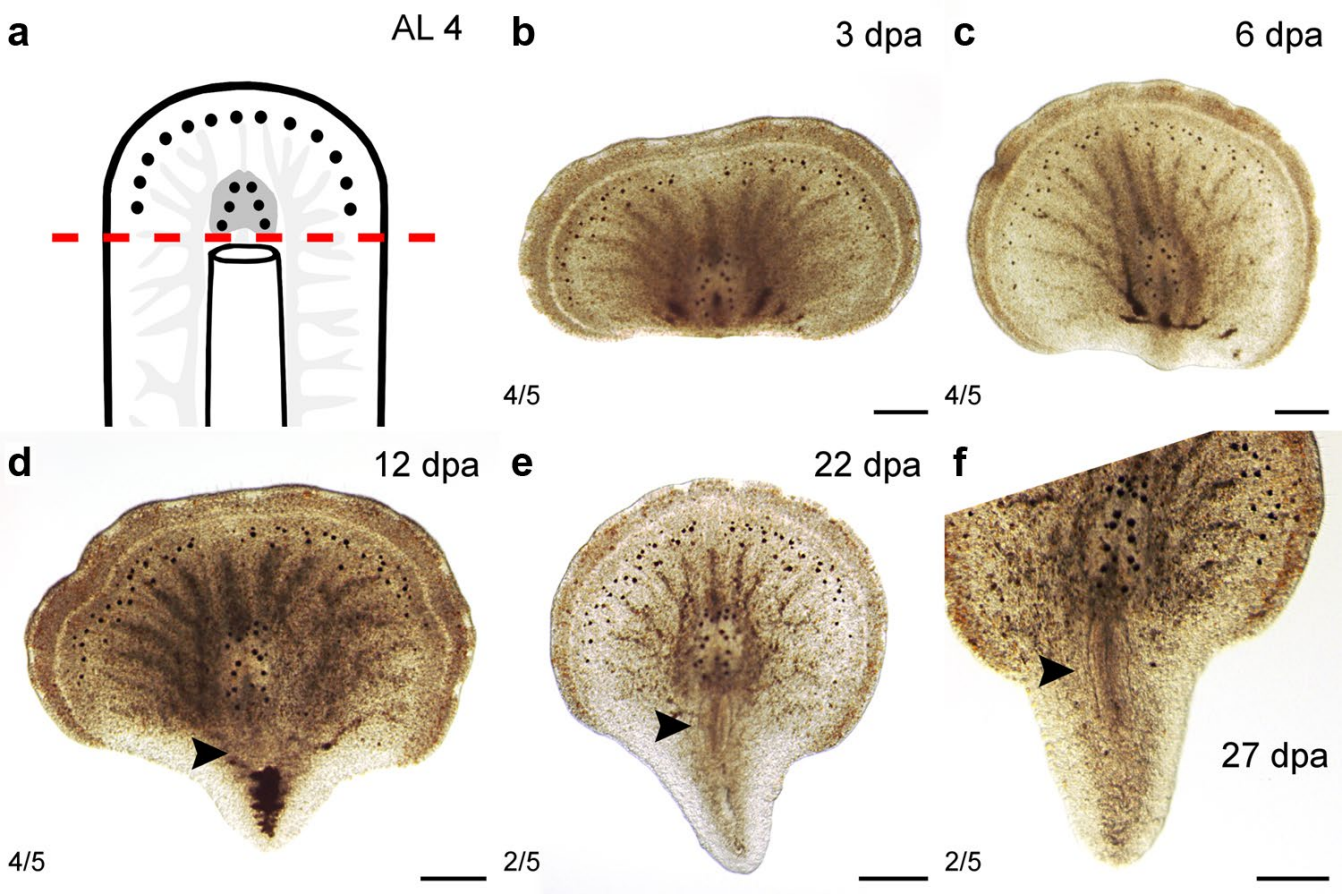
Regenerates of AL 4—right behind the brain. **a** Scheme with AL 4. **b**–**f** Posterior regeneration of AL 4. **b** The posterior regenerates 3 dpa; the blastema was starting to form. **c** At 6 dpa, the tip of the tail started reforming in the regenerating tissue. **d** After 12 dpa, a regenerated pharynx (arrowhead) started to become visible. **e**, **f** Between 22 and 27 dpa, the pharynx had grown in size and was already visible. Scale bars: 250 µm

The posterior regenerates of AL 4 (*n *= 15) contained the entire brain and all tissues in front of the brain (Fig. 5a). They were capable to regenerate a pharynx and a steadily regrowing tail plate (Fig. 5b–f). Thus, they did not form a circular shape as the posterior regenerates of AL 2 with amputations in front of the brain and AL 3 horizontally through the brain did. Neither reproductive organs nor a sucker became visible during the observation period of 45 days.

**Fig. 6:**
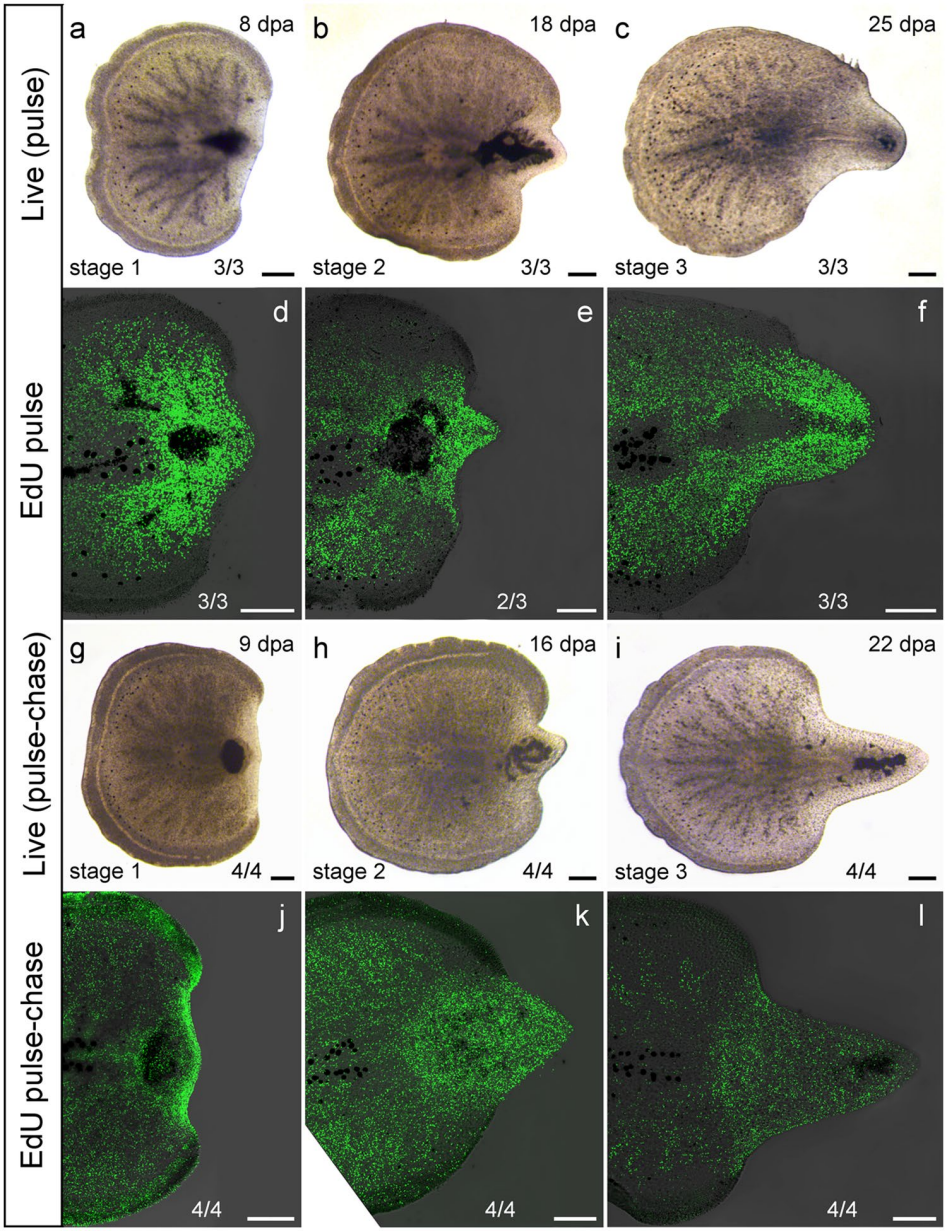
Pulse and pulse-chase experiments of pharynx regenerates (AL 4 posterior regeneration). **a**–**c** Live images of stage 1–3 specimens of the pulse experiment on the day of fixation. **d-f** Fixed EdU-labelled specimens of stages 1–3. Stages 1 and 2: high accumulation of stained cells inside and near the blastema, none in the epidermis and area of the brain. Stage 3: many stained cells in the blastema, none in the epidermis and newly regenerated pharynx. **g**–**i** Live images of stage 1–3 specimens of the pulse-chase experiment on the day of fixation. **j**–**l** Fixed EdU-labelled specimens of stages 1–3. In all stages the highest abundance of stained cells was found in the blastema and EdU labelling in all types of cells, including epidermal tissues. Scale bars: 200 µm

The posterior regenerates kept at 15 °C (*n *= 9) formed a blastema 8 ± 0.3 dpa (Fig. 6a, g) and 15 ± 3 dpa, the intestine became visible in the regenerating tissue (Fig. 6b, h). The specimens reformed a visible pharynx 24 ± 4 dpa (Fig. 6c, i; Table 3).

The posterior regenerates kept at 20 °C (*n *= 6) formed a blastema 3 dpa (Fig. 5b), which was formed into a pointed tip 6 ± 3.8 dpa (Fig. 5c). A pharynx regenerated 12 ± 4.2 dpa (Fig. 5d–f; Table 3). In these specimens, the contents of the intestine remaining from prior to the amputation had been digested and excreted 22 dpa (Fig. 5d, e).

In the anterior regenerates, the pharynx, gut, sucker and genital pores remained. The regenerates of both temperature settings were not able to regenerate a head (Suppl. Figure 1a, d). At both temperatures, a blastema was formed 6 dpa and the wound was closed. Pigmentation of the regenerated tissue occurred about 12 dpa (data not shown). Within 4 weeks, all anterior regenerates died without regenerating any of the missing organs (Table 4).

#### Amputation through the pharynx (AL 5)

**Fig. 7:**
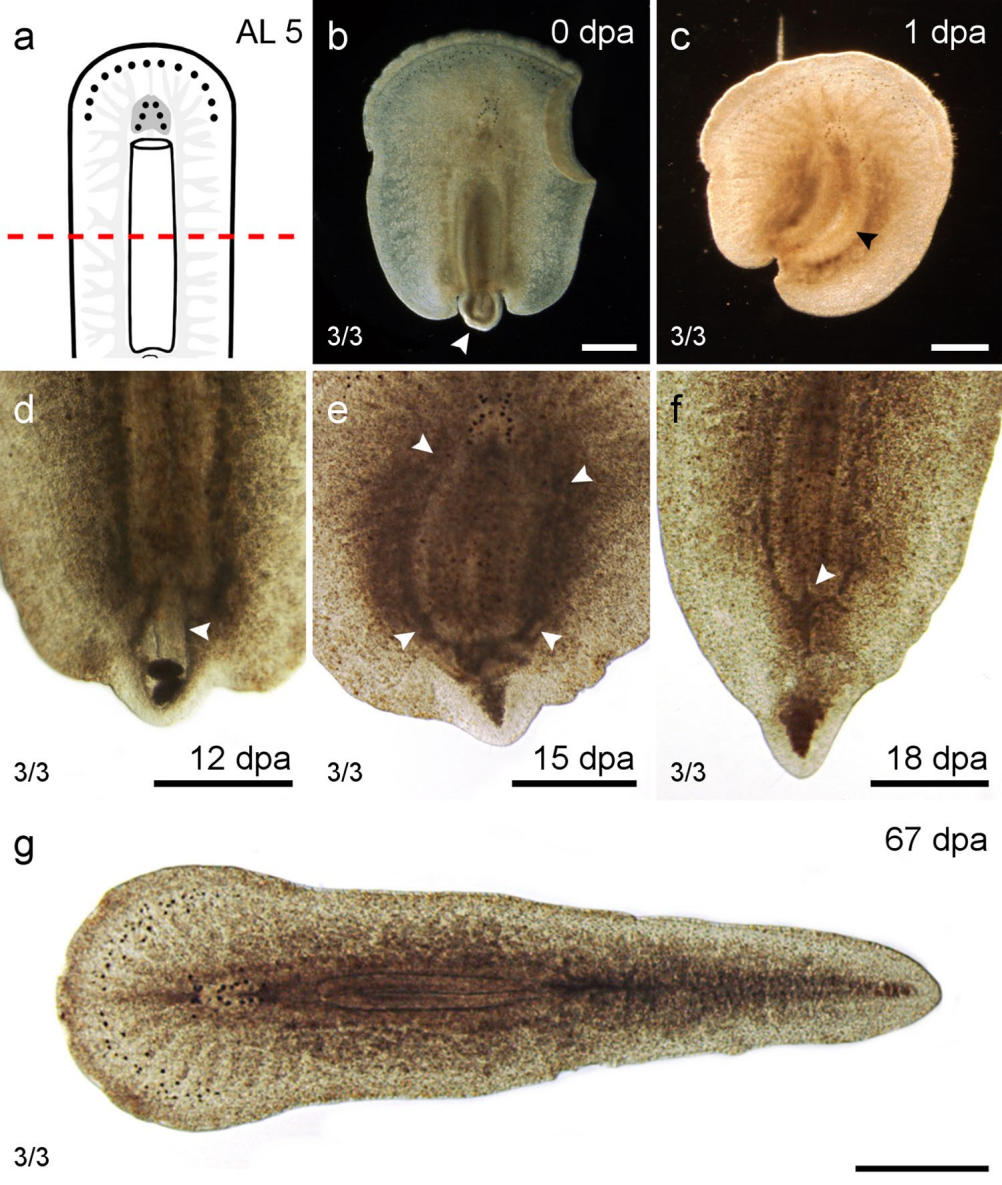
Regenerates of AL 5—through the pharynx. **a** Scheme with AL 5. **b**–**g** Posterior regeneration of AL 5. **b** The posterior regenerate on the day of amputation. The anterior half of the pharynx remained inside the pharynx sheath (arrowhead). **c** One day post amputation, the pharynx had been discarded and the pharynx sheath was empty (arrowhead). **d** The pharynx started to reform after 12 days inside the pharynx sheath (arrowhead). **e** After 15 dpa, the pharynx had grown in size and the dark undigested particles seen in **d** had been discarded. **f** After 18 dpa, the ‘Darmmund’ (arrowhead) became visible. **g** After 67 days, the animal looked similar to before amputation. Scale bars: 500 μm

Amputations were made through the pharynx (*n *= 3), with half of it remaining in the posterior and the other half remaining in the anterior regenerate (Fig. 7a). The posterior regenerates discarded the disconnected pharynx tube 1 dpa (Fig. 7b, c). Within 6 dpa, the blastema formed a pointed tip and showed pharynx regeneration 12 ± 4.2 dpa (Fig. 7d, e; Table 3). The entrance of the intestine, the so-called Darmmund (Bresslau 1933), became visible 18 dpa (Fig. 7f) and 67 dpa, the animal had restored the original proportions (Fig. 7g).

The anterior regenerates discarded the remaining pharynx after 1 week. Wound closure was observed but anterior regenerates were neither able to regenerate a head nor any of the missing organs such as the discarded pharynx (Suppl. Figure 1b, e; Table 4).

#### Amputation just posterior of the pharynx (AL 6)

**Fig. 8:**
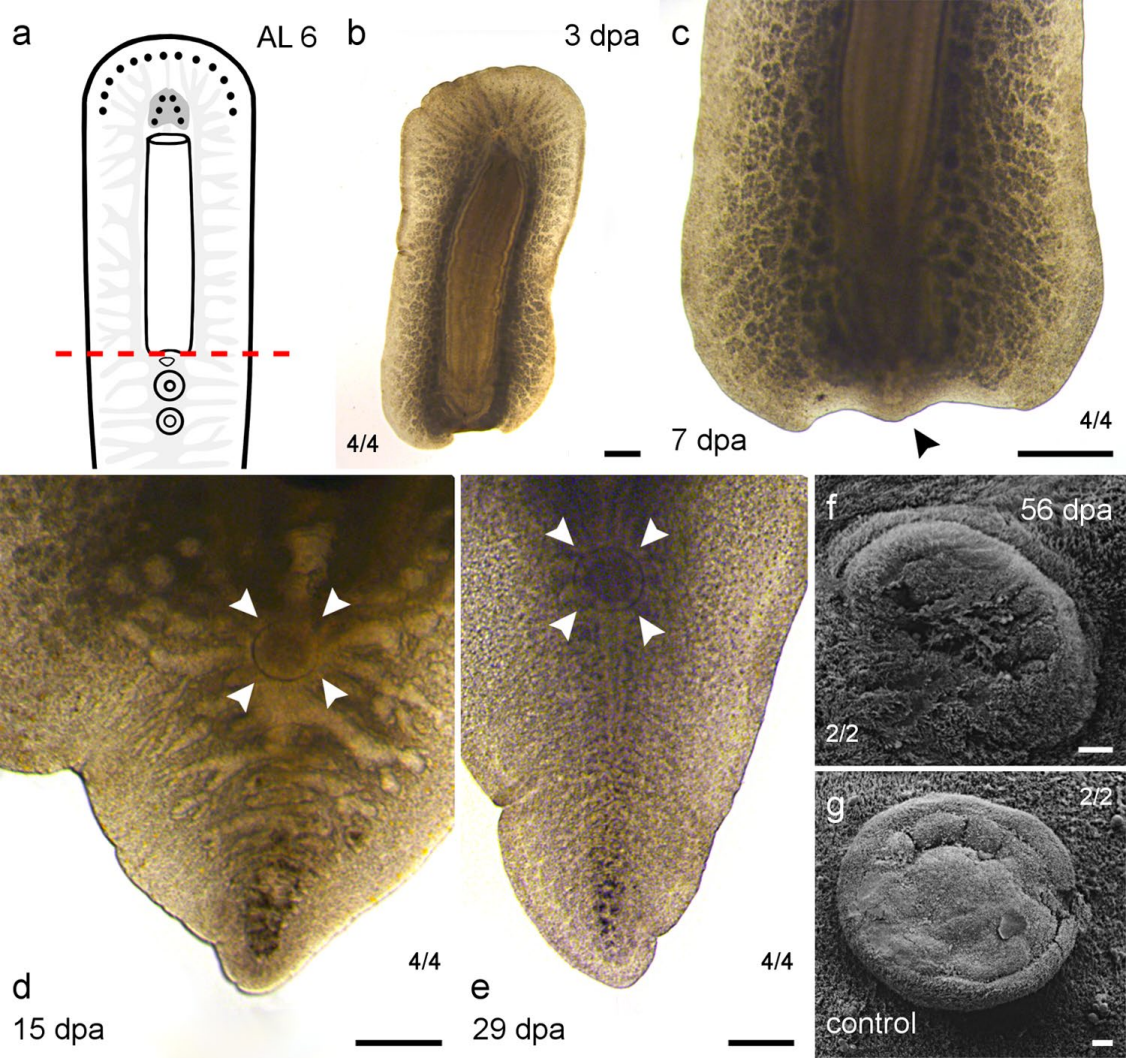
Regenerates of AL 6—right behind the pharynx. **a** Scheme with AL 6. **b**–**e** Posterior regeneration of AL 6. **b** Posterior regenerate 3 dpa. **c** At 7 dpa, the tail tip (arrowhead) started to grow back. **d** The regenerated and fully functional sucker (arrowhead) was visible 15 dpa. **e** After 29 dpa, the tail had grown in size, the main branch of the gut had reformed and the sucker is visible (arrowhead). **f** SEM picture of the regenerated sucker 56 dpa of the same individual seen in **b**–**e**. **g** SEM picture of a sucker from an intact animal. Scale bars: **b**, **c** 500 µm, **d**, **e** 200 µm, **f**, **g** 20 µm

The posterior regenerates of AL 6 (*n *= 4) were missing all structures behind the pharynx, including the genital pores and the sucker (Fig. 8a, b). A blastema with a small tipped tail was visible 7 dpa (Fig. 8c). All specimens were able to regenerate a functional sucker 15 dpa (Fig. 8d; Table 3). Two of the regenerated suckers were more closely observed under a scanning electron microscope (SEM) 56 dpa (Fig. 8f) and compared with two non-regenerating control animals (Fig. 8g). Regenerated and control suckers were of similar size but the depicted control sucker was fixed in a more protruded position than the regenerated sucker, which is also partially covered by surrounding epidermal cilia (Fig. 8f, g). A tail was well regenerated 29 dpa (Fig. 8e). The genital pores were not detected to regenerate within the observation period of 2 months.

The anterior regenerates (*n *= 4) were missing all structures in front of the genital pores (Fig. 8a). None of these structures could be regenerated. Wound closure was observed but no further regeneration could be detected (Table 4).

#### Amputation posterior of reproductive organs and sucker (AL 7)

**Fig. 9:**
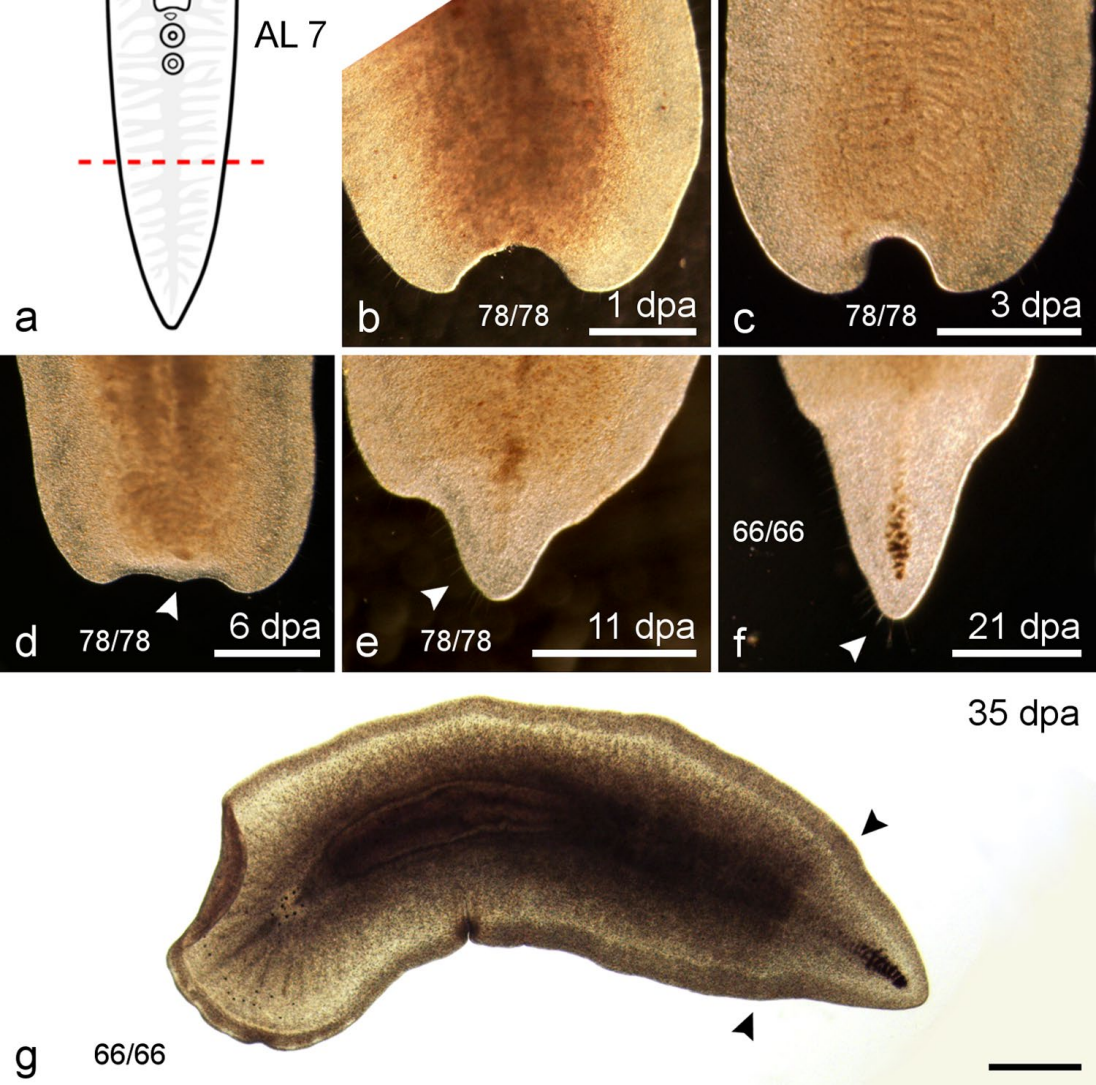
Regenerates of AL 7—behind the genital pores and the sucker. **a** Scheme with AL 7—behind the genital pores and the sucker. **b**–**g** Posterior regeneration of AL 7. **b** The posterior regenerate 1 dpa. **c** Three days post amputation, the wound was closed and the blastema started to form. **d** After 6 days, a tip (arrowhead) became visible in the regenerating blastema. **e** After 11 dpa, regrown long sensory cilia (arrowhead) had been observed. **f** The tail tip had grown largely in size by 21 dpa and long sensory cilia are visible (arrowhead). **g** After 35 days, the regenerated tissue was still visible due to lighter pigmentation (arrows). The images in **b**–**g** do not show the same individual. Scale bars: 500 µm

**Fig. 10:**
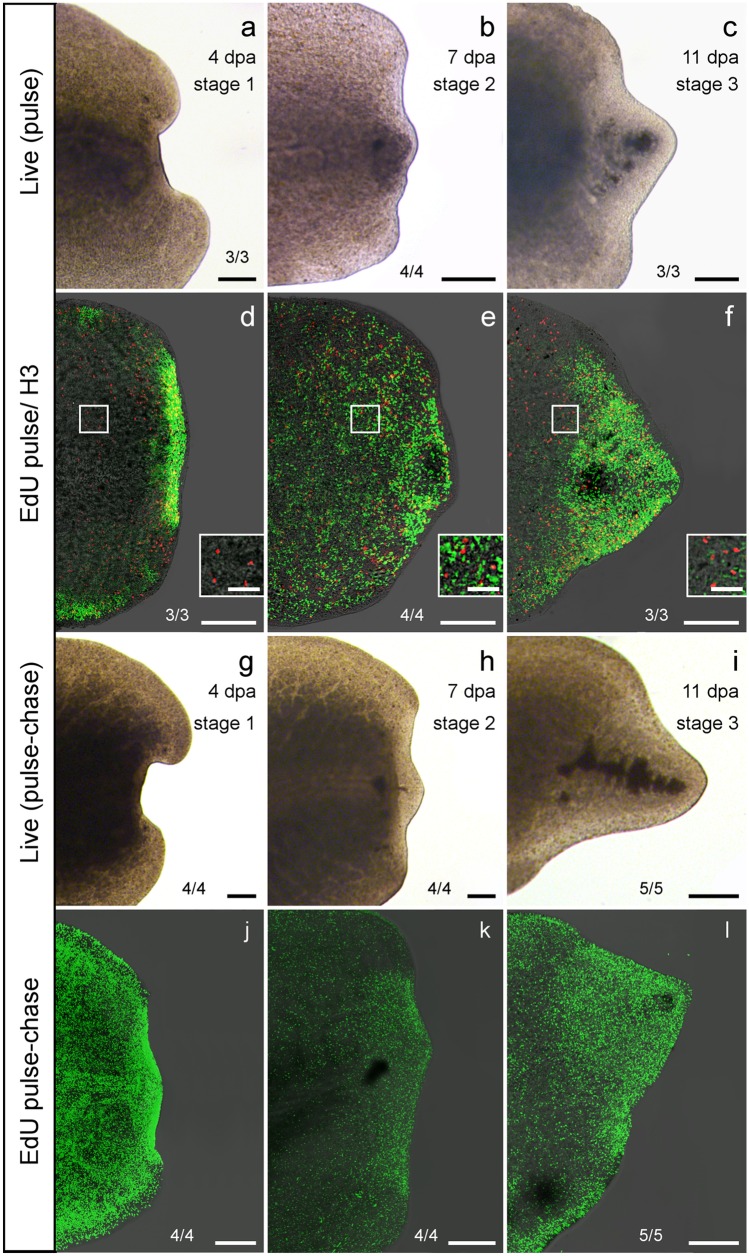
Pulse and pulse-chase experiments of tail plate regenerates (AL 7 posterior regeneration). **a**–**c** Live images of stage 1–3 specimens of the pulse experiment on the day of fixation. **d-f** Fixed EdU/pH3-labelled specimens of stages 1–3. No stained cells were found in the epidermis of specimens of any stage. **d** Stage 1: high abundance of EdU-labelled cells in regeneration blastema and areas near the body wall, except epidermis. Mitotic cells awere stained throughout all visible tissues except epidermis. **e** Stage 2: most S-phase cells were stained in the regeneration blastema. S-phase and mitotic cells were found in all shown tissues except epidermis. **f** Stage 3: Most S-phase cells were found in the regeneration blastema with a slight gradient towards its bordering tissues. Mitotic cells were found throughout all shown tissues except epidermis. **d**–**f** Insets show proliferating cells in higher magnification. **g**–**i** Live images of stages 1–3 specimens of the pulse-chase experiment on the day of fixation. **j**–**l** Fixed EdU-labelled specimens of stages 1–3. In all three stages labelled cells were found in all cell types. **j** Stage 1: after 4 days the highest density of stained cells was found in the regeneration blastema. A lot of labelled cells could be found in the intact tissues as well. Labelled cells could be found in all cell types. **k** Stage 2: after 7 days, most labelled cells were found in the regeneration blastema and bordering tissue. **l** Stage 3: at 11 dpa, most labelled cells were found in the regenerated tissue. Scale bars: 200 µm, except insets 50 µm

The regenerates of this cutting level (Fig. 9a) were kept at 15 °C (*n *= 12) and at 20 °C (*n *= 66). At 1 dpa and 3 dpa, the wound site had a concave shape (Fig. 9b, c). Regardless of temperature in all posterior regenerates, the blastema started to form a pointed tip 6 ± 3.8 dpa (Figs. 9d and 10b, h; Table 3), which became a visible tail tip after about 8 days (data not shown). Within 10 ± 0.9 dpa, the tail was reformed and long sensory cilia were visible in animals kept at 15 °C and 20 °C (Figs. 9e, f and 10c, i). After 35 days, the tail plate of regenerates kept at 20 °C had returned to its original shape and size but pigmentation remained less dense in the regenerating tissue and made it possible to distinguish the regenerated tissue from the darker nonregenerated tissue (Fig. 9g). Animals kept at 15 °C were used for staining experiments to observe stem cell dynamics and were all fixed 11 dpa.

The anterior regenerates, which consisted of the tail plate (Fig. 9a), were not able to regenerate any missing organs (Suppl. Figure 1c,f). Wound closure was observed as well as the formation of a small blastema. From this point, no further regeneration occurred. This was true for regenerates kept at both temperatures (Table 4).

#### Amputation longitudinally through the entire animal (AL 8)

All specimens (*n *= 3) of longitudinal amputations through the entire worm (Fig. 1b) dispersed within 2 days (Table 2).

### Stem cell dynamics

In intact animals, proliferating cells (S-phase cells and cells in mitosis) could be found throughout the entire animal, even in front of the brain. However, The epidermis and the area of the pharynx were devoid of proliferating cells. The inside of the gut showed no proliferation but neoblasts could be found in the walls of the main trunk and branches (Fig. 1c–e).

### Pharynx regenerates

To observe stem cell dynamics, sections at AL 4 right behind the brain were repeated. The regenerates were kept at 15 °C. Cells were then EdU-labelled to either chase the fate of proliferating cells labelled right before amputation to specific regeneration stages with a pulse-chase experiment or to observe S-phase cell distributions in certain regeneration stages with a pulse experiment.

After amputations were made right behind the brain regeneration stage 1 (*n *= 7) was reached by the posterior regenerates after 8–9 dpa, when regenerating tissue became visible (Fig. 6a, g). Stage 2 (*n *= 7) was reached once the intestine became visible in the regenerating tissue after 15 ± 3 dpa (Fig. 6b, h). Stage 3 (*n *= 7) was reached as soon as a newly regenerated pharynx became visible after 24 ± 4 dpa (Fig. 6c, i).

In the regenerates of the pulse experiments, proliferating cells were found throughout the entire animal except for the epidermis, which was completely devoid of proliferating cells and the area of the brain with only very few stained cells. A strong aggregation of EdU-labelled cells was observed in the regeneration blastema. This was true for all three regeneration stages (Fig. 6d–f). In stage 1 (*n *= 3), inside the regeneration blastema an area started to form, where dark, undigested particles accumulate and only very few S-phase cells were stained (Fig. 6d). Once stage 2 (*n *= 3) was reached 10 days later, that same area had expanded, with no stained cells in its centre (Fig. 6e). In this area, the new pharynx was then formed and was clearly visible in stage 3 (*n *= 3) specimens. No stained cells were found in the area of the newly regenerated pharynx (Fig. 6f).

In the pulse-chase stainings labelled cells could be found in all cell types including ectodermal tissues like pharynx and epidermis (Fig. 6j–l). Stage 1 (*n *= 4) specimens (Fig. 6j) showed a higher abundance of stained rhabdites—gland cells located in the epidermis and used for protection—than stages 2 (*n *= 4) and 3 (*n *= 4) (Fig. 6k, l). These stained rhabdites are likely background staining as both the shape of the label (rod-like) and the localisation in the epidermis suggest that gland vesicles are stained and not the (in-sunk) nuclei. In stage 3, the area of the pharynx showed stained cells (Fig. 6l). In all three stages, the density of stained cells was highest in the regeneration blastema (Fig. 6j–l).

None of the negative controls (*n *= 2) showed EdU-specific staining but unspecific staining of the conically shaped rhabdites could be observed (Suppl Figure 2).

### Tail plate regenerates

Another series of pulse and pulse-chase experiments was made with specimens that had their tail plate—the posterior third of their body—removed (AL 7). The tail plate regenerates were fixed using specific time points. Stage 1 (*n *= 7) was reached after 4 days (Fig. 10a, g), stage 2 (*n *= 8) after 7 days (Fig. 10b, h) and stage 3 (*n *= 8) after 11 days (Fig. 10c, i).

In pulse specimens of stage 1 (*n *= 3), only the regeneration blastema and tissues close to the body wall showed a high abundance of EdU-labelled S-phase cells. Mitotic cells were stained throughout all visible tissues except epidermis and pharynx, with a slightly higher density in the regeneration blastema (Fig. 10d). In pulse specimens of stage 2 (*n *= 4), the highest density of S-phase cells was also found in the regeneration blastema. S-phase and mitotic cells were found in all shown tissues except epidermis (Fig. 10e). In pulse specimens of stage 3 (*n *= 3), S-phase cells were found in high abundance in the regeneration blastema with a slight gradient towards its bordering tissues. Mitotic cells were found throughout all shown tissues. No stained cells were found in the epidermis (Fig. 10f).

In the pulse-chased specimens of stage 1 after 4 days (*n* = 4), the highest density of stained cells was found in the regeneration blastema. EdU-labelled cells can be found in all cell types, including epidermis (Fig. 10j). In stage 2 animals (*n* = 4), most stained cells were found in the regeneration blastema and bordering tissue. Stained cells were also found in the epidermis (Fig. 10k). In stage 3 specimens (*n* = 5), the border of not yet pigmented regenerated tissue (Fig. 10i) was also visible in the form of an especially high density of stained EdU-labelled cells (Fig. 10l). None of the negative controls (*n* = 5) showed EdU-specific stainings but a slight background and stained rhabdites were visible (data not shown).

## Discussion

### Regeneration capacity of *P. siphunculus*

All regenerates with their brain remaining intact were able to regenerate most of the missing tissues and organs, including anterior structures such as the submarginal and cerebral eyes and posterior structures such as gut, pharynx and sucker. Genital pores were not observed to regenerate. This could be due to missing or very slow gonad regeneration after amputation as animals were considerably smaller after the observed regeneration period without feeding.

With half a brain remaining in the regenerates, the results varied. Posterior regenerates with amputations made horizontally through the brain were not capable to regenerate missing organs. In each case, these posterior regenerates formed a circular shape upon wound closure and then ceased to regenerate any further. The anterior regenerates were able to regenerate cerebral and submarginal eyes.

Bigger regenerates that had the lateral half of their brain removed were able to regenerate submarginal and cerebral eyes but the missing brain tissue was not observed to regenerate. While submarginal eyes were also regenerated on the side of the missing brain half, cerebral eyes only appeared in close proximity to the existing brain tissue and never in a pattern that resembled control animals. In triclads, the brain induces eye regeneration and eyes cannot regenerate in the absence of the brain (Brøndsted 1969). Interestingly, in the polyclad *T. mediterranea*, some eyes were regenerated even in the complete absence of the brain if cut just posterior of the brain (Bertemes et al. 2020). This indicates that also (nerve) cells outside the brain capsule can induce eye regeneration, at least in *T. mediterranea*. In *P. siphunculus*, the remaining half of the brain can induce submarginal eye regeneration in the newly regenerating half of the head.

Only two polyclad species (*Thysanozoon brocchi* and *Leptoplana tremellaris*) have been reported to be able to regenerate their lateral brain half in previous studies. However, these findings were not supported by histological sections or stainings (Monti 1900; Child ; 1905a). With *P. siphunculus*, we did not find lateral brain regeneration, though further studies with histological sections and stainings should be made to ascertain these results.

The acotylean *Notocomplana humilis* was found to regenerate the anterior half of the brain (Ishida 1998) as did the cotylean *Theama mediterranea* (Bertemes et al. 2020). To date, *T. mediterranea* remains the sole cotylean shown to regenerate the anterior half of its brain as we were not able to observe brain regeneration after horizontal cuts through the brain of *P. siphunculus*.

It was stated by the ‘rule of polyclad regeneration’ that anterior regeneration is not possible if the brain is completely removed (Olmsted 1922). This was also true for *P. siphunculus*. None of the anterior regenerates with no brain remaining was able to regenerate further upon wound closure. In these regenerates, no missing organs were observed to reform during the observation period.

Posterior regenerates without a brain or only half a brain were also not able to regenerate. Since EdU-labelled cells were shown to be abundant in the area in front of the brain (Fig. 1c), the number of proliferating cells should not be the issue in this case, as it was for the catenulid *Paracatenula galateia* in another study (Dirks et al. 2012). One possible explanation could be that the amount of remaining tissue in the regenerate was too small. To test this hypothesis, further experiments need to be made to evaluate the minimal number of cells needed for regeneration in *P. siphunculus*, as has been done for the triclad *Dugesia dorotocephala* (Montgomery and Coward 1974) or *Macrostomum lignano* (Egger et al. 2006). Other than the size, the brain tissue missing in these anterior pieces may be key to their lack of regeneration, as hypothesised by Child (1910) and Bertemes et al. (2020).

If a cut was made vertically through the entire animal, all specimens died within 2 days. This was most likely due to the large wound site which could not easily be minimised by contracting the bordering tissue.

### Regeneration categories

There are four categories defined for polyclad regeneration: (I) no regeneration is possible except for small peripheral parts; (II) regeneration of organs posterior to the brain but no observed anterior regeneration; (III) regeneration of all organs except the complete brain regeneration of most organs in the absence of the brain; (IV) as group III but additionally able to regenerate the anterior part of the brain (Bertemes et al. 2020).

*P. siphunculus’* congener *Prosthiostomum dohrni* was found to be able to regenerate posterior structures such as a pharynx in another study (Lang 1884) and was therefore placed in group II. *P. siphunculus* does not fit into group II, because it is capable to regenerate anterior structures such as submarginal and cerebral eyes in specimens of AL 2 and 3 (Figs. 3g–i and 4e–i). As it can regenerate most organs in the presence of at least half the brain but is not able to regenerate brain tissue, it fits best into group III, even though it cannot regenerate missing organs in the absence of the brain as the other two cotylean species can, which also belong to this group: *Thysanozoon brocchi* (Monti 1900; Levetzow 1939) and *Eurylepta cornuta* (Dalyell 1853).

### Temperature

Temperature conditions influenced the speed of organ regeneration in the pharynx regenerates of AL 4. Posterior regenerates amputated right behind the brain took twice as long to regenerate a pharynx at 15 °C as their counterparts at 20 °C. The significance of temperature on regeneration speed was previously addressed in a study on *M. lignano*, in which regenerates were also found to regenerate faster at higher temperatures (Wudarski et al. 2019). The influence of temperature on regeneration is not limited to flatworms but has also been observed in vertebrates such as newts and zebrafish (Schauble and Nentwig 1974; Sîrbulescu and Zupanc 2010). The findings on the posterior regenerates of AL 4 of *P. siphunculus* also show this effect on regeneration at lower temperatures. However, no difference in regeneration speed was observed between posterior regenerates of AL 7 kept at higher (20 °C) and lower (15 °C) temperature. This suggests that 10 ± 0.9 days was the minimum time needed to regenerate the tail plate.

### Irregular behaviour after removal of the lateral half of the brain

Irregular feeding behaviour after decerebration, excision of half the brain or inversion of the brain after transplantation is known from *Notoplana acticola* (Koopowitz et al. 1976; Koopowitz and Holman 1988; Davies et al. 1985). In one of these studies on *N. acticola*, one lobe of its brain was removed and the severed nerves grew back together with the remaining half of the brain. However, regular response behaviour as was observed prior to amputation was only resumed to 60% in these animals (Faisst et al. 1980). In another series of experiments with *N. acticola*, the effects of brain inversions were tested. To do so, the entire brain was removed. Then, these brains were transplanted into another host animal, which also served as a donor. The brain was then transplanted in four different orientations in these hosts: a transplant with regular orientation as control, with the dorsal and ventral side inverted, anterior and posterior reversed, dorsal and ventral side inverted and anterior and posterior reversed. Control animals resumed regular behaviours including feeding behaviour. Animals with brain inverted, reversed and reverse inverted brains resumed regular behaviours, except for feeding behaviour (Davies et al. 1985).

In the present study, *P. siphunculus* also showed irregular feeding behaviour after one of its brain lobes was removed and was not able to resume regular feeding behaviour within the observation period of 4 months. Future experiments with brain lobe excisions on *P. siphunculus* should be combined with staining methods such as FMRFamide or serotonin to test its neuronal repair abilities.

### Stem cell distribution

Neoblast stem cells were never found in the ectodermal tissues of studied rhabditophoran flatworms. S-phase cells were only ever found in mesodermal tissues such as parenchyma (Egger et al. 2009b). In the present study, we show that this is also true for *P. siphunculus*.

Several studies on planarians, macrostomorphans, catenulids and proseriates revealed the brain as the anterior border for proliferation (Newmark and Alvarado 2000; Nimeth et al. 2007; Dirks et al. 2012; Girstmair et al. 2014). In the polyclad, *T. mediterranea* stem cells are distributed similarly: two rows of proliferating cells, one on each side ranging from the tip of the tail up to the brain (Bertemes et al. 2020). In *P. siphunculus*, however, EdU pulse stainings of intact animals revealed the presence of proliferating cells throughout the entire animal, including the area anterior of the brain. Only ectodermal tissues showed no stained cells. 

No staining was detected in the area of the brain which most likely shows a lack of stem cells in that region (Fig. 6d–f). However, this lack of stained cells could also result from the impermeable brain capsule (see Bertemes et al. 2020).

In pulse specimens, no proliferating cells could be found within the epidermis or area of the pharynx. A small region with only very few proliferating cells could be observed in the pulse specimens of pharynx regenerates after 8 days. That same region expanded and later grew into a small regenerated pharynx (Fig. 6d–f). According to these results, pharynx primordia formation probably starts after about a week post amputation.

Pulse specimens of the tail plate regenerates also showed no proliferating cells inside the epidermis. However, there was also an area in the middle of the body, which showed much fewer EdU-labelled cells for stage 1 and 3 specimens (Fig. 10d, f). The fact that tissues close to the body wall were stained and a gradient was visible coming from the blastema suggests that one of the staining components did not enter as well with these specimens. The issue might have been that the tail plate was cut off above the newly regenerated tissue before fixation. Upon wounding, the animal might have ejected mucous, which made it harder for the staining components to later enter through the epidermis. However, mitotic cells were stained in these areas and suggest that proliferation occurs at a normal rate in the middle of the body.

### Proliferation in blastema

The intensely studied Tricladida show no or very little proliferation in their regeneration blastema. Proliferation usually takes place at the border of the wound site and new undifferentiated cells migrate from there inside of the blastema (Saló and Baguñà 1984; Morita and Best 1984). Among flatworms, this only seems to be the case in triclad species. In all other free-living flatworms, which have been studied so far, proliferating cells were found inside their regeneration blastema (Egger et al. 2009a; Dirks et al. 2012; Girstmair et al. 2014; Bertemes et al. 2020). For example in the macrostomorphan *M. lignano*, cells were found to proliferate rapidly within the regeneration blastema (Egger et al. 2009a). Similarly, *P. siphunculus* showed a high density of proliferating cells within its regeneration blastema in the pulse experiments (Figs. 6d–f and 10d–f). The highest density of the stained cells was found inside the regenerating tissue. This shows that a high number of proliferating cells were recruited for the regeneration process right after the amputations were made.

### Stem cell migration

In the pulse-chase experiments, stained cells were found in all cell types including the pharynx primordia. Similar results have been found for pharynx regeneration in the proseriate *Monocelis* sp. or the polyclad *T. mediterranea*, which also showed no proliferating cells inside the pharynx primordia but pulse-chased cells were shown to migrate into the newly regenerated pharynx (Girstmair et al. 2014; Bertemes et al. 2020). In the macrostomorphan *Macrostomum lignano*, pulse-chased cells were found to migrate into the genital primordium but to not proliferate there anymore (Egger et al. 2009a).

In the pharynx regenerate that was chased for almost a month (Fig. 6), the regenerated tissue shows a higher density of stained cells, likely because most S-phase cells labelled prior to amputation had migrated to the wound site and kept proliferating to regenerate the lost tissues.

## Conclusions

We found that *P. siphunculus* fits best into group III of the four established groups of polyclad regeneration. Members of group III are capable to regenerate all organs except the complete brain and to regenerate most organs in the absence of the brain. *P. siphunculus* is capable to regenerate anterior structures and not able to regenerate its brain which is why it fits in neither group II (no regeneration of anterior structures) nor group IV (regeneration of the brain). So even though it cannot regenerate most organs in the absence of the brain it fits best into group III.

We were able to show that proliferating cells are abundant in the parenchyma but completely absent in ectodermal tissues and organ primordia. Proliferating cells were even found anterior of the brain which is not the case for other flatworms. In regenerates, proliferating cells were found inside the blastema, as was previously found in all studied flatworms, except in the Tricladida. Our findings further support the hypothesis that the proliferation-free blastema is an apomorphy of the Tricladida.

## Ethical approval

All applicable international, national and/or institutional guidelines for the care and use of animals were followed.

## Electronic supplementary material

Below is the link to the electronic supplementary material.

Supplementary file1 (TIFF 3532 kb)

Supplementary file2 (TIFF 2543 kb)

## Abbreviations

ASW: Artificial sea water
BSA-T_x_: PBS-T_x_ with 1% bovine serum albumin
DAPI: 4′,6-Diamidino-2-phenylindole
Dpa: Days post amputation
EdU: 5-Ethynyl-2′-deoxyuridine
FA: Formaldehyde
oN: Overnight
PBS: Phosphate-buffered saline
PBS-T_x_: PBS with 0.1% Triton X-100
pH3: Phosphorylated histone H3
RT: Room temperature
