# Supplementary figures and images for: Regeneration of the flatworm Prosthiostomum siphunculus (Polycladida, Platyhelminthes)
Source: Cell Tissue Res. 2020 Nov 7;383(3):1025–41. doi: 10.1007/s00441-020-03302-w (PMC7960593; doi:10.1007/s00441-020-03302-w)

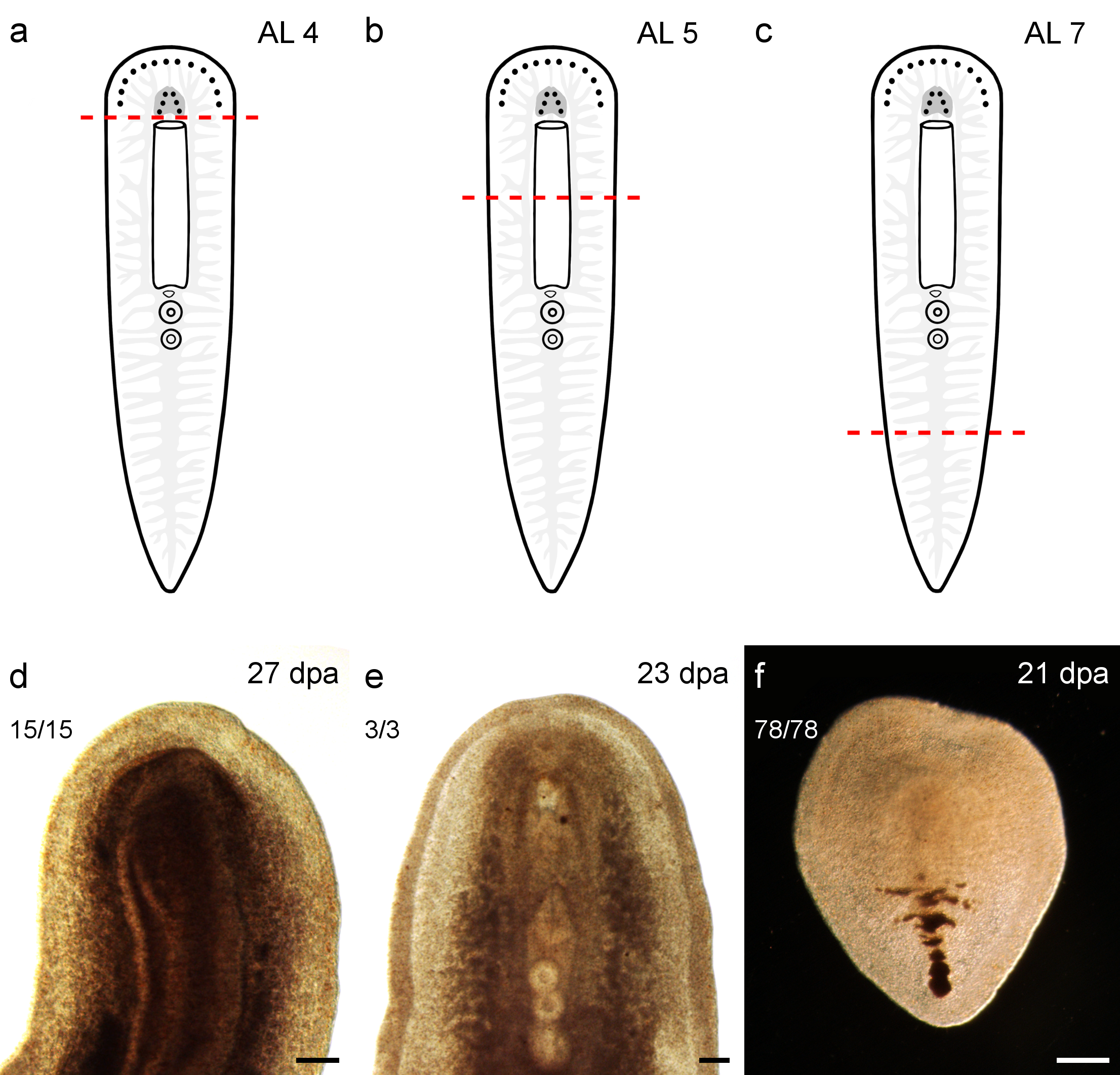

Supplement: Supplementary file 1 — Supplementary file1 (TIFF 3532 kb) [file 441_2020_3302_MOESM1_ESM.tiff]

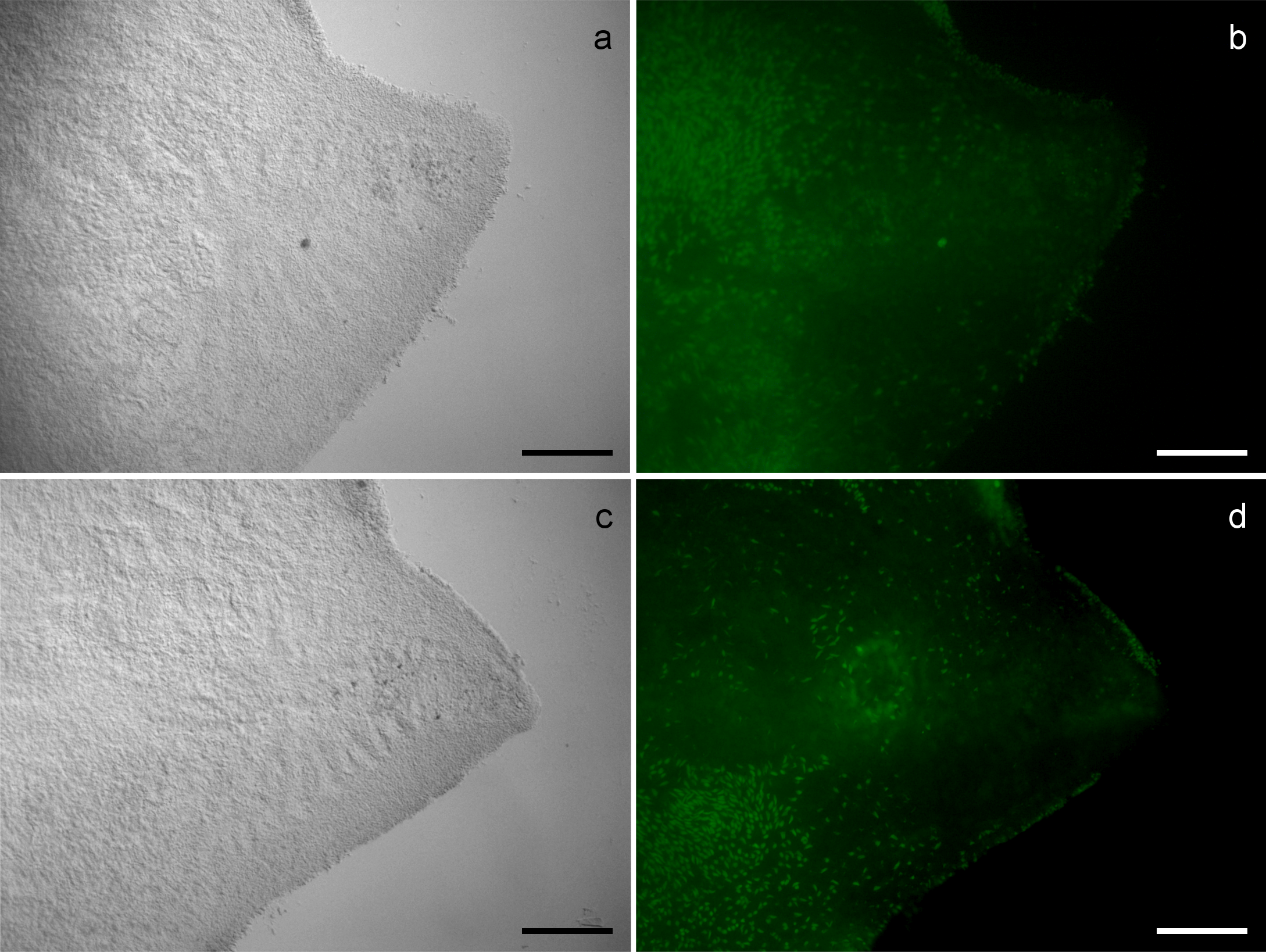

Supplement: Supplementary file 2 — Supplementary file2 (TIFF 2543 kb) [file 441_2020_3302_MOESM2_ESM.tiff]
